# Change in Indications and Outcomes for Stereotactic Biopsy Following Transition from Full Field Digital Mammography + Digital Breast Tomosynthesis to Full Field Synthetic Mammography + Digital Breast Tomosynthesis

**DOI:** 10.3390/medsci13010029

**Published:** 2025-03-12

**Authors:** Jose Net, Antoine Hamedi-Sangsari, Taylor Schwartz, Mirelys Barrios, Nicole Brofman, Cedric Pluguez-Turull, Jamie Spoont, Sarah Stamler, Monica Yepes

**Affiliations:** Department of Radiology, Leonard M. Miller School of Medicine, University of Miami, Miami, FL 33136, USA; axh1583@med.miami.edu (A.H.-S.); taylor.schwartz@jhsmiami.org (T.S.); mirelys@med.miami.edu (M.B.); nbrofman@med.miami.edu (N.B.); cedricpluguez@miami.edu (C.P.-T.); jrosenkrantz@med.miami.edu (J.S.); s.stamler@med.miami.edu (S.S.); myepes@med.miami.edu (M.Y.)

**Keywords:** digital breast tomosynthesis, synthetic mammography, stereotactic biopsy

## Abstract

Background: Synthetic 2D mammography was developed to decrease radiation exposure, but to our knowledge there have been no studies evaluating the impact of implementation of full field synthetic mammography/digital breast tomosynthesis (FFSM/DBT) on indications for stereotactic biopsy. Objective: To compare indications and biopsy outcomes for stereotactic biopsy for full field digital mammography (FFDM/DBT) to those of FFSM/DBT. Methods: Retrospective chart review of stereotactic biopsies performed from July 2014 to September 2018. Reports were reviewed and indication for biopsy, lesion size, and final pathology were recorded. Comparison between the two groups following transition to FFSM/DBT in 2016 was performed. Results: 66 of 361 stereotactic biopsies performed in the FFDM/DBT group were malignant (PPV 18.3%), compared to 60 of the 391 biopsies performed in the FFSM/DBT group (PPV 15.4%) with no significant difference in PPV (*p* = 0.281). There were statistically significant changes in indications for biopsies after transitioning to FFSM/DBT: with a decrease in calcifications referred for biopsy (68.03% vs. 89.75%; *p* < 0.001), and a statistically significant increase in referral of masses (10.74% vs. 4.43%; *p* < 0.001), asymmetries (15.60% vs. 5.26%; *p* < 0.001), and architectural distortion (5.63% vs. 0.55%; *p* < 0.001). PPV across all indications (21.8% in FFSM/DBT vs. 20.3% in FFDM; *p* = 0.213), and invasive cancer yield (5.63% vs. 3.32%; *p* = 0.129) remained comparable following transition to FFSM/DBT without statistically significant differences. Conclusions: Following transition to FFSM/DBT, statistically significant shifts in indications for biopsies were observed with a decrease in referral of calcifications and an increase for masses, asymmetries and architectural distortions. PPV for stereotactic biopsy was not significantly different and cancer yield across all indications remained similar, with an increase in invasive cancer diagnosis.

## 1. Introduction

Digital breast tomosynthesis (DBT) was approved in 2011 by the United States Food and Drug Administration (FDA) to be used in combination with standard full-field digital mammography (FFDM) for breast cancer screening [1]. The addition of DBT to FFDM increases breast cancer detection rate, especially for invasive cancers, and decreases recall rates in the screening setting when compared to FFDM alone [2,3,4,5,6,7,8,9,10]. The idea of combining FFDM with DBT stemmed from the concern that subtle findings, such as calcifications, could be missed by DBT alone [11]. However, combined FFDM/DBT effectively doubles the radiation dose received by the patient [11]. To address the issue of higher radiation exposure, a synthetic 2D image was developed. The synthesized 2D image is created utilizing information from the DBT dataset and thus eliminates the need for the additional FFDM acquisition [12]. The elimination of the acquired 2D image not only reduces radiation exposure with levels comparable to FFDM alone, it also decreases the time that patients are in compression, which reduces patient motion artifacts [13,14,15,16]. FFSM gained FDA approval in 2013. Since then, many studies have demonstrated equivalent performance of FFSM/DBT compared with FFDM/DBT [4,17,18,19].

One of the initial concerns when transitioning from the FFDM to synthesized mammography, was the potential limited visualization of calcifications mostly secondary to the lower resolution of FFSM compared to FFDM. Dodelzon et al. compared diagnostic performance of FFDM and FFSM in the detection of microcalcifications and found no difference in measures of diagnostic accuracy, sensitivity, NPV, and PPV between FFSM and FFDM [20]. Durand et al. demonstrated that even though FFSM has lower resolution than FFDM, cancer detection was preserved [14]. Kilic et al. also demonstrated similar diagnostic performance of FFSM compared to FFDM in the evaluation calcifications, regardless of reader experience level [21].

To our knowledge there have been no studies evaluating the impact of implementation of FFSM/DBT on indications for stereotactic biopsy. This study aimed to assess whether the transition to FFSM/DBT altered the indications for stereotactic biopsy and to determine its impact on cancer detection rates and biopsy outcomes.

## 2. Methods and Materials

This retrospective study is compliant with the Health Insurance Portability and Accountability Act (HIPAA) and was approved by our human subjects institutional review board. The requirement to obtain informed consent was waived. No monetary support from outside sources was received.

All patients who underwent diagnostic work up and subsequent stereotactic core needle biopsy at Sylvester Comprehensive Cancer Center from July 2014 to September 2018 were included in the study. This time period was chosen given our practice’s transition into FFSM/DBT in the fall of 2016, and included two years in which patients underwent FFDM/DBT prior to their biopsies (July 2014 to August 2016) and two years in which patients underwent FFSM/DBT prior to their biopsies (September 2016 to September 2018). The hospital electronic medical record (Epic Systems, Madison, WI, USA) and PACS (Phillips, Andover, MA, USA) at our institution were used to access radiology reports, pathology reports, and mammographic images. The indications (calcifications, mass, asymmetry, and architectural distortion), lesion size, and final pathology were recorded.

### 2.1. Imaging Technique

All mammograms were acquired with the same imaging systems (Selenia Dimensions 2D/3D system, Hologic, Marlborough, MA, USA) which included craniocaudal (CC) and mediolateral oblique (MLO) projections. SM images were generated from the DBT images by using Food and Drug Administration (FDA)-approved, commercially available processing software (C-view 1, Hologic Inc., Marlborough, MA, USA).

### 2.2. Statistical Analysis

Descriptive statistics and chi square test of association were used to compare the two groups (FFDM/DBT versus FFSM/DBT). The indications (calcifications, masses, asymmetries, and architectural distortions) referred for biopsy were compared among the FFDM and FFSM groups. Our two cohorts consisted of the stereotactic biopsies recommended two years before and after the transition to FFSM/DBT at the end of August 2016. The positive predictive value (PPV): defined as the number of true positive biopsies/number of biopsies, across the two groups and across various indications (calcifications, mass, and asymmetry) were also compared. Positive predictive value of architectural distortions among the two groups were not compared due to the small sample size, which would not yield a meaningful comparison due to lack of statistical power. The final histologic finding was classified as malignant in cases of DCIS and/or invasive carcinoma on the stereotactic core biopsy or on final surgical excision. Overall breast center metrics were reviewed for patients imaged at our main cancer center, including cancer detection rate across all indications for mammography (screening and diagnostic), PPV3 for all resultant biopsy recommendations regardless of guidance, and interval cancer rates (defined as cancers that develop between routine mammograms) were compared among the FFDM and FFSM groups. Tests of statistical significance were two-sided, with *p* less than 0.05 considered indicative of a statistically significant difference. All statistical analyses were performed using IBM SPSS Statistics, version 28.0 software (Armonk, NY, USA).

## 3. Results

**Figure 1:**
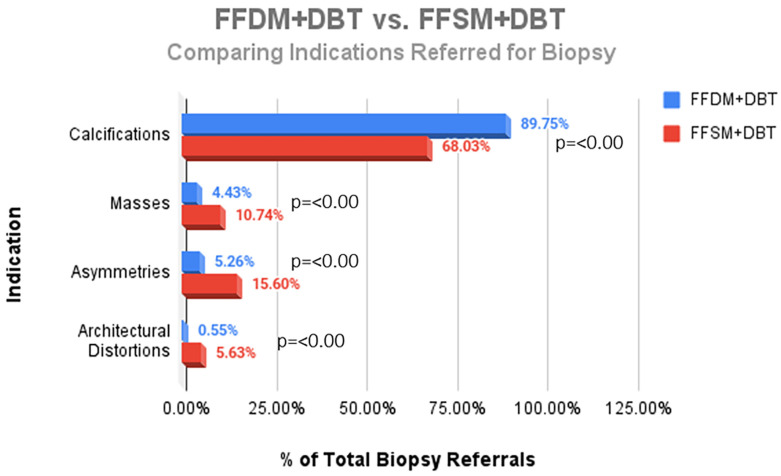
Comparison of the proportion of different indications referred for stereotactic core needle biopsy in the FFDM + DBT vs. the FFSM + DBT cohorts.

**Figure 2:**
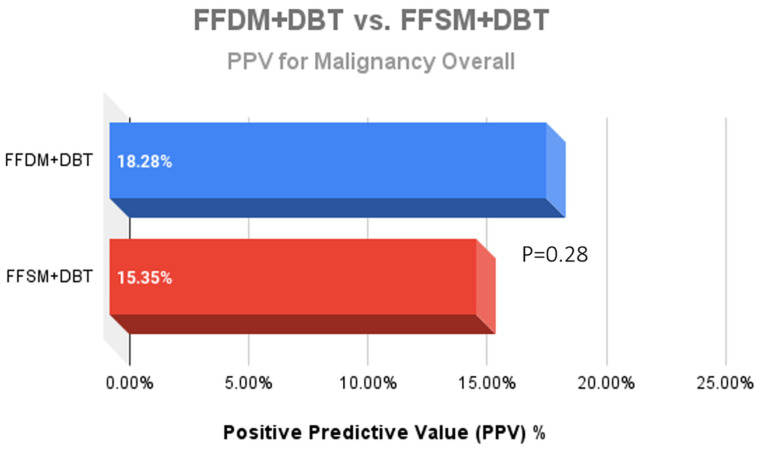
Comparison of the Positive predictive value (PPV) outcomes for malignancy after 361 stereotactic biopsies with FFDM + DBT vs. 391 stereotactic biopsies with FFSM + DBT demonstrated no significant difference between the two groups.

A total of 752 stereotactic biopsies from July 2014 through September 2018 were included in this study. There were 361 stereotactic biopsies in the FFDM/DBT cohort from July 2014–August 2016 and 391 stereotactic biopsies in the FFSM/DBT cohort from September 2016–September 2018. No significant differences were observed for the overall PPV for malignancy in the FFDM/DBT vs. FFSM/DBT cohorts (18.28% vs. 15.35%; *p* = 0.281). When comparing indications for stereotactic biopsy in FFDM/DBT vs. FFSM/DBT (Figure 1), there was a statistically significant decrease in referral for calcifications in the FFSM cohort (68.03% vs. 89.75% *p* < 0.001), and statistically significant increase in referrals for masses (10.74% vs. 4.43%; *p* < 0.001), asymmetries (15.60% vs. 5.26%; *p* < 0.001), and architectural distortion (5.63% vs. 0.55%; *p* < 0.001). Despite these differences, the PPV for malignancy was not statistically significantly different between FFDM/DBT vs. FFSM/DBT across all indications as follows (Figure 2): calcifications (18.7% vs. 17.7%; *p* = 0.711), masses (15.2% vs. 14.5%; *p* = 0.925) and asymmetries (5.3% vs. 6.6%; *p* = 0.839). PPV differences could not be measured for architectural distortions due to a small sample size, which did not achieve statistical power.

**Figure 3:**
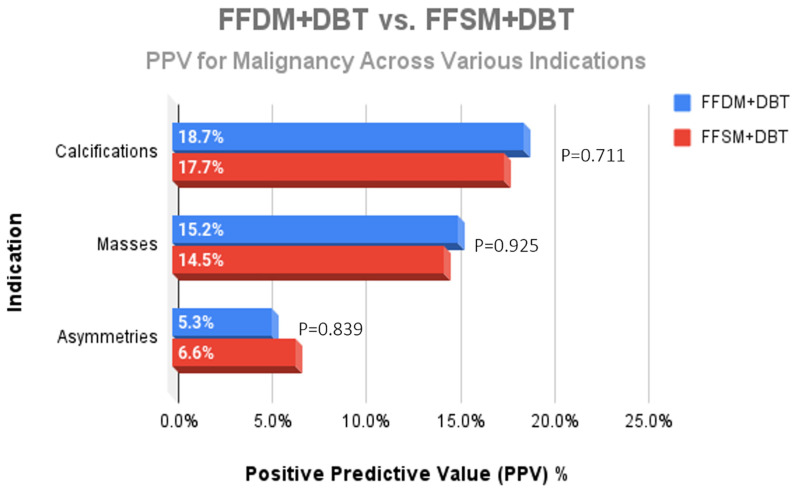
Comparison of the PPV for malignancy across the different indications for stereotactic core needle biopsy revealed no significant difference when comparing lesions identified on FFDM + DBT and FFSM + DBT.

**Figure 4:**
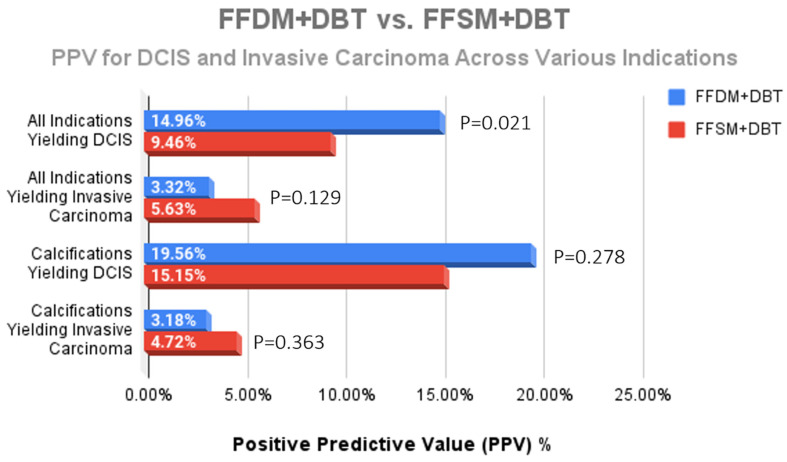
Comparison of the PPV for DCIS and invasive carcinoma across all indications for stereotactic biopsy. The only statistically significant difference was a decreased DCIS yield across all indications; however, no significant difference for DCIS nor invasive cancer yield due to calcifications was observed following transition to FFSM + DBT.

While a statistically significant decrease for the overall diagnosis of DCIS was observed after implementation of FFSM/DBT (14.96% FFDM/DBT vs. 9.46% FFSM/DBT; *p* = 0.021) (Figure 3), there was no significant difference in DCIS yield from calcifications (19.56% vs. 15.15%; *p* = 0.278) (Figure 4). Although we observed a trend toward increase in invasive cancer detection rates with an 70% increase in the FFSM/DBT group across all indications (5.63% vs. 3.32%; *p* = 0.129) and a 48% increase in invasice cancer yield from biopsies performed for microcalcifications (4.72% vs. 3.18%; *p* = 0.363), these were not statistically significant. When reviewing overall cancer center metrics prior to and following the transition to FFSM/DBT, statistically significant improvement was observed for cancer detection rate (21.1/1000 in FFSM/DBT vs. 16.4/1000 in FFDM/DBT; *p* < 0.001), yet no significant differences were observed in the PPV3 rates between the two groups (21.8% in FFSM/DBT vs. 20.3% in FFDM; *p* = 0.213) or in the interval cancer rate (1.197/1000 in FFSM/DBT vs. 1.370/1000 in FFDM/DBT; *p* = 0.491) (Table 1).

## 4. Discussion

The purpose of this study was to determine if there was a significant change in indications for stereotactic guided biopsy when comparing FFDM/DBT (2014–2016) to FFSM/DBT (2016–2018) and to review implications on outcomes. We found statistically significant differences across all indications for stereotactic biopsy, with a decrease in referral of calcifications for biopsy and increase in referrals for masses, architectural distortions, and asymmetries following transition to FFSM/DBT. We attribute the increased proportion of masses, asymmetries, and distortions referred for sterotactic biopsy to the availability of tomosynthesis biopsy capability in our system in 2016.

The transition to FFSM/DBT has historically been controversial due to concerns regarding limited evaluation of microcalcifications with FFSM, which is particularly relevant in evaluating indications for stereotactic guided biopsy. Our study is in line with findings by Spangler et al., who found a higher sensitivity for the detection of calcifications with FFDM compared to DBT: (0.84% [95% CI, 0.79–0.88%]) vs. (0.75% [95% CI, 0.70–0.80%]) [22] and consistent with the TOMMY trial in which a comparable cancer detection rate was obtained despite a slight decrease in the detection of malignant calcifications (85% vs. 88%) but with better specificity: (44% vs. 39%) [23]. Our results are also similar to those of Choi et al. who retrospectively evaluated calcification conspicuity in 198 patients and found no significant difference between FFSM and FFDM alone or with DBT, suggesting that FFSM with DBT is sufficient in diagnosing calcifications without FFDM [24]. Moreover, Zuley et al. found that performance levels as demonstrated by probability of malignancy–based area under the receiver operating characteristic curve (AUC) analysis with FFSM alone (AUC = 0.89) and in combination with tomosynthesis (AUC = 0.92) were comparable to performance levels when using DM alone (AUC = 0.89) or DM with tomosynthesis (AUC = 0.94), concluding that two-dimensional FFSM can be used as an acceptable replacement for directly acquired mammograms in tomosynthesis-based evaluations [25].

While we did observe a significant drop in the number of stereotactic biopsies for calcifications (*p* < 0.001) following transition to FFSM/DBT, our data demonstrate that there was no statistically significant difference between the FFDM and FFSM groups when looking at DCIS or invasive cancer yield from calcifications (*p* = 0.278 and 0.363, respectively). Advances in technology, such as the implementation of FFSM, have improved calcification conspicuity and reduced the possibility of missing malignant calcifications at screening [26,27]. Although there was a statistically significant drop in DCIS yield for all stereotactic biopsies (*p* = 0.021) following the transition to FFSM/DBT, the PPV for malignancy for calcifications was maintained in the FFSM group. We surmise that the drop in DCIS yield overall was largely due to the observed shift in indications with larger numbers of asymmetries, masses, and architectural distortions referred for biopsy in the 2016–2018 cohort, which are more likely to result in an invasive cancer diagnosis.

Our data reflects a trend towards increased invasive cancer yield following transition to FFSM/DBT, although this was not statistically significant. We observed a 70% increase in invasive cancer diagnosis overall in the FFSM group, including a 48% increase in invasive cancer yield when performing biopsies for calcifications (*p* = 0.129 and *p* = 0.363, respectively). This data is in line with the TOSYMA trial, the largest randomized controlled trial to date to assess DBT/FFSM, which showed that the invasive cancer detection rate was 48% higher with FFSM/DBT compared to FFDM alone [10]. It is important to highlight that following transition to FFSM/DBT, we obtained significant increases in cancer detection (21.1/1000 in FFSM/DBT vs. 16.4/1000 in FFDM/DBT; *p* < 0.001) with a similar PPV3 (21.8% in FFSM/DBT vs. 20.3% in FFDM/DBT; *p* = 0.213) and similar interval cancer rates for our center (1.197/1000 in FFSM/DBT vs. 1.370/1000 in FFDM/DBT; *p* = 0.491). 

## 5. Limitations

Limitations of our study include that this is a single institution, retrospective study, which limits generalizability and the ability to establish causality. Our results may have differed in other settings with different patient demographics, but our results represent that of a major metropolitan city with diverse patient population that includes a large proportion of Hispanic patients adding to available literature in this population. This was not a reader study and so did not account for differences in calcification detection rates. The trend towards increased specificity of calcifications referred for biopsy in the FFSM/DBT group and lack of significant changes in interval cancer rates support previous studies confirming non-inferiority, such as Dodelzon et al., that confirmed no significant difference in detection rates when comparing FFDM to FFSM in their reader study [20,21,22,23,24,25]. Our study did not account for changes in radiologist experience which may influence our results. While the analysis included comparison of biopsy outcomes, interval cancer rates, and overall cancer detection rate metrics, long term follow up data was not available which could have allowed for more insight into the effectiveness of FFSM/DBT in cancer detection. While we note the benefit of reduced radiation exposure, our study did not evaluate the definite reduction in radiation dose to illustrate the impact of this benefit. The small number of architectural distortions in our study limited their evaluation due to lack of statistical power.

## 6. Conclusions

Our study confirms that breast radiologists can expect significant differences in indications for stereotactic biopsy following transition to tomosynthesis based screening and adds to the growing body of literature that FFSM/DBT is an effective tool for the detection and characterization of breast abnormalities. The transition from FFDM/DBT to FFSM/DBT should be considered a safe and effective solution to reduce radiation exposure without loss of sensitivity and potentially improved specificity particularly where it pertains to invasive and biologically active disease. Furthermore, the decrease in exposure time and radiation may enhance patient satisfaction and adherence to screening recommendations.

## Figures and Tables

**Table 1:** Comparison of overall cancer center metrics including outcomes for all patients presenting for breast imaging in our main cancer center including screening and diagnostic imaging as well as biopsy outcomes for all guidance modalities. (CDR = Cancer Detection Rate, ICR = Interval Cancer Rate).

Comparing Overall Cancer Center Metrics			
	FFDM + DBT	FFSM + DBT	*p*-Value
CDR	16.4/1000	21.1/1000	<0.001 ***
PPV3	20.3%	21.8%	0.213
ICR	1.370/1000	1.197/1000	0.491

## Data Availability

The original contributions presented in this study are included in the article. Further inquiries can be directed to the corresponding author.
